# Understanding the Psychosocial Effects of WES Test Results on Parents of Children with Rare Diseases

**DOI:** 10.1007/s10897-016-9958-5

**Published:** 2016-04-20

**Authors:** Lotte Krabbenborg, L. E. L. M. Vissers, J. Schieving, T. Kleefstra, E. J. Kamsteeg, J. A. Veltman, M. A. Willemsen, S. Van der Burg

**Affiliations:** 1 Radboud Institute for Health Sciences, Radboud University Medical Center, Nijmegen, the Netherlands; 2 Institute for Science, Innovation and Society (ISIS), Radboud University, P.O. Box 9010, 6500 Nijmegen, the Netherlands; 3 Department of Human Genetics, Donders Centre for Neuroscience, Radboudumc, Geert Grooteplein 10, 6525 Nijmegen, the Netherlands; 4 Department of Paediatric Neurology, Radboud University Medical Center, Nijmegen, the Netherlands; 5 Department of Clinical Genetics, Maastricht University Medical Centre, Universiteitssingel 50, 6229 Maastricht, the Netherlands

**Keywords:** Whole exome sequencing, Genetic counselling, Parental experiences, Rare diseases, Psychosocial

## Abstract

The use of whole exome sequencing (WES) for diagnostics of children with rare genetic diseases raises questions about best practices in genetic counselling. While a lot of attention is now given to pre-test counselling procedures for WES, little is known about how parents experience the (positive, negative, or inconclusive) WES results in daily life. To fill this knowledge gap, data were gathered through in-depth interviews with parents of 15 children who underwent WES analysis. WES test results, like results from other genetic tests, evoked relief as well as worries, irrespective of the type of result. Advantages of obtaining a conclusive diagnosis included becoming more accepting towards the situation, being enabled to attune care to the needs of the child, and better coping with feelings of guilt. Disadvantages experienced included a loss of hope for recovery, and a loss by parents of their social network of peers and the effort necessary to re-establish that social network. While parents with conclusive diagnoses were able to re-establish a peer community with the help of social media, parents receiving a possible diagnosis experienced hurdles in seeking peer support, as peers still needed to be identified. These types of psychosocial effects of WES test results for parents are important to take into account for the development of successful genetic counselling strategies.

## Introduction

Parents of children with rare genetic diseases such as intellectual disability, neurodegenerative disorders, and/or epileptic encephalopathies, typically spend years searching for a diagnosis, causing emotional turmoil (Carmichael et al. 2015; Graungaard and Skov 2006). This extensive search for a diagnosis is sometimes termed a “diagnostic odyssey,” that is, parents bring their children to different specialists, where they are subjected to myriad examinations and tests (Carmichael et al. 2015). Despite all of these tests, a definitive diagnosis is reached in less than 50 % of cases. The introduction of Next Generation Sequencing (NGS) technologies, allowing geneticists to examine the entire genome or the protein-coding part of the genome, the exome, in a single test, is considered promising as NGS increases the chance of finding a disease-causing mutation. Recent studies have confirmed that Whole Exome Sequencing (WES) increases the amount of diagnoses for children with hitherto unexplained developmental delay (De Ligt et al. 2012; Iglesias et al. 2014; Vissers et al. 2010). Therefore, WES is increasingly used as a first-tier diagnostic test in clinical practice for clinically and genetically heterogeneous disorders.

This diagnostic development raises questions, however, about genetic counselling strategies. Currently much attention is given to the information that should be provided in the pre-test counselling procedure prior to the child’s (and parents’) participation in WES trials, such as information on informed consent and the possibility of incidental findings (Appelbaum et al. 2014; Krabbenborg et al. 2015; Rigter et al. 2013; Hitch et al. 2014; Levenseller et al. 2013; Roche and Palmer 2014; Sapp et al. 2013; Tabor et al. 2012). Little is known, however, about how parents process and experience a (positive, negative, or inconclusive) result from WES when they return home to their daily lives with their child. To provide adequate psychosocial support and sufficient information in pre- and post-counselling related to WES diagnostics, it is critically important for providers to know about these parental experiences. Such knowledge will allow genetic counsellors and other medical specialists to anticipate disappointments and questions that WES results may raise for parents. The purpose of this study was to investigate these psychosocial aspects. Specifically, we explored how a diagnosis impacts daily life by asking parents what they perceive as the benefits and the disadvantages they experienced after receiving WES results.

## Methods

### Diagnostic Exome Sequencing Process

This research is part of a multidisciplinary translational study that took place at the Radboud University Medical Center (Radboudumc), involving the departments of Human Genetics, Pediatric Neurology, and Health Evidence, as well as the Institute for Quality of Healthcare. Between September 2011 and March 2012, 50 children (<18 yrs) with complex pediatric neurological problems of presumed genetic origin were included. They presented with clinically and genetically heterogeneous disorders, such as intellectual disability, movement disorders, epileptic seizures, muscle disorders, and/or speech disorders. Diagnostic sequencing was performed as described in a previous study (De Ligt et al. 2012), and diagnostic outcomes were communicated to parents over the course of 2013 and 2014. Three types of diagnostic reports were issued: a definitive genetic diagnosis, referring to a situation in which a pathogenic mutation was encountered in a known disease gene explaining the phenotype of the patient (*n* = 13; Group 1); a possible diagnosis, referring to a situation where a (likely) pathogenic variant was identified in a gene not yet associated with disease (*n* = 16; Group 2); or no genetic diagnosis (*n* = 21; Group 3). This study was approved by the medical ethics committee of the Radboud University Medical Center under the realm of diagnostic exome sequencing.

### Participants and Data Collection

To assess the value of WES test results for the daily life of parents, we conducted in-depth, semi-structured interviews, preferably face-to-face at parents’ homes which allowed for observation of the family’s daily life. In some cases, interviews were conducted by telephone. Respondents were recruited via the pediatric neurologists (JS, MW) involved in our translational study. Once verbal consent of parents was obtained, the interviewer (LK) called the parents to make an appointment for the interview.^1^ In total, interviews were conducted with parents of 15 children: 10 couples and 6 single parents. Six of the 15 patients received a definitive diagnosis by WES (group 1), including Kabuki syndrome (*de novo* mutation *KDM6A*), MECP2 duplication syndrome (maternally inherited X-linked duplication), 16p microdeletion syndrome (*de novo* deletion), congenital Rett syndrome (*de novo* mutation *FOXG1*), autosomal dominant mental retardation 5 (*de novo* mutation *SYNGAP1*), and a *de novo* mutation in a novel candidate gene leading to intellectual disability (*PHIP*). Five of the 15 patients received the message that a lead for a possible genetic diagnosis was identified, yet needed further confirmation (group 2). In four patients no genetic cause was identified (group 3). No incidental findings were reported in the patients and interviewees (i.e., an unexpected finding unrelated to the medical condition the patient is receiving the sequencing for but of medical importance for patient care). The interviewees lived in the Eastern and Southern parts of the Netherlands, and all but one were of Caucasian origin.

A semi-structured interview protocol was developed which included twenty open questions and probes as this tactic allowed us to gain a rich insight into parents’ experiences. The protocol covered two main topics: 1) parents’ evaluation of the WES-counselling procedure, including questions concerning motivations, informed consent, and incidental findings, and 2) parents’ evaluation of whether and how the WES test results changed their daily life with their child. This paper primarily addresses parents’ responses to questions concerning how WES impacted their daily lives (2), for instance: “Can you describe what it means to live with a child with a medical condition?” “Can you describe your day-to-day activities?” “Do you connect with peers (parents in similar situations)?” “Can you describe whether and how the WES test results influenced your already-established relations with peers?” (For more information on parents’ evaluation of the WES counselling protocol, see Krabbenborg et al. 2015.) The aim of the interviews was to gain insight into a variety of themes. The interviews were carried out one at a time and when no new themes arose, and thus saturation was achieved, we ended our data collection (Evers 2007). The interviews lasted approximately 1–1.5 h.

For groups 1 and 3, interviews were scheduled within 2 months after receiving the results, whereas for group 2 these took place within 2 to 6 months after receiving the diagnosis. In addition, additional telephone interviews were conducted after 6 months with four of six parents in group 1 to allow us to compare longer-term experiences, specifically between having a conclusive diagnosis and a possible diagnosis. Follow-up interviews with parents from group 3 were not initiated as we anticipated not much had changed in their situation.

### Data Analysis

The interviews were recorded and transcribed verbatim. The transcripts were uploaded in Atlas-ti 7.1. By using a content analysis approach (Boeije 2012; Vaismoradi et al. 2013), a coding scheme was developed that reflected central themes articulated by parents. Researcher LK and SvdB independently coded first transcripts to inductively ascribe codes. Final consensus on the coding scheme was reached through discussion. Examples of codes are “confirmation of being a responsible parent” (definitive diagnosis) and “feelings of isolation” (definitive and possible diagnosis).

## Results

Throughout the interviews we found that all families who live with a child with a (presumed) genetic disease engage in similar activities as each other. These activities can be seen as the interpretative framework that shapes parental experiences with the child as well as their assessment of WES test results, and can be classified into three categories:

- seeking knowledge about the child’s condition and prognosis;
- caregiving activities and management of care; and
- finding a supportive environment.

We will describe these core activities and show how WES results influence them. Illustrative quotes are presented in text boxes and have been translated from Dutch.

### Seeking Knowledge about the Child’s Condition and Prognosis

Parents living with a child with a rare, presumably genetic disease are often confronted with uncertainties because they lack information about the condition, prognosis, therapy and recurrence risk of the disease. To meet this uncertainty, or as a coping mechanism, parents try to gather as much information as possible; for example, by visiting one medical specialist after another (cf. the “diagnostic odyssey”), by searching the internet, but also by exchanging information with other parents with children with similar complaints via social media and through participating in patient organizations. With WES, parents hoped to obtain a diagnosis, which would allow them to gather more information from all sources listed above.

When receiving WES test results, parents from all the three groups (definite diagnosis, possible diagnosis, and no diagnosis) experienced relief as well as worries. Parents who received a definitive diagnosis appreciated having a confirmation of their suspicion that their child’s condition truly had a medical cause, and was not a result of medical malpractice during birth or parental carelessness or deficient upbringing. Parents also valued the fact that the diagnosis provided more insight into the inheritance and recurrence risks. On the other hand, receiving a definitive diagnosis implied a loss of hope for some parents. While parents participated in WES because they wanted to know the cause of the child’s condition, our data suggest that some parents hoped more information about their child’s disease would lead to better treatment, and even hoped for some miraculous recovery to happen. For some of the parents the diagnosis was disappointing because it pointed towards a syndrome, and they considered “a syndrome” as something that cannot be cured, in contrast to “a disease.” Other parents receiving a definitive diagnosis were relieved because it pointed to a better prognosis than initially thought. Yet, they were also disappointed because their health care providers knew little to nothing about their child’s disease. In response to this problem, some parents took control; they collected the information about the disease and distributed it to other (local) health care specialists to inform them about the child’s diagnosis and the medical condition itself.

Parents receiving a “possible diagnosis” responded to it with ambivalence: on the one hand, they continued the search for a definitive diagnosis (hope), and on the other hand they made efforts to come to terms with the situation (acceptance). Additionally, in specific cases where a maternally inherited X-linked recessive mutation was mentioned as the potential cause of disease (requiring further familial segregation studies), the possible diagnosis generated led to new worries, uncertainties, and insecurities.

Parents who received no diagnosis varied in their responses. Some were relieved that nothing was found in the genes of their child, but some were also concerned because they still longed for an explanation of their child’s symptoms.

Text box 1: Quotations illustrating that WES test results evoke ambivalent responses

**Table:** 

Longing for a conclusive diagnosis	“The neurologist called and told us that they did not find a diagnosis. And to be honest, that was disappointing. I know this may sound strange, because on the one hand you do not want to hear that there is something wrong with your child, but on the other hand, she is still diseased.” (no diagnosis, interview 8) “On the one had I am glad that they did not find something in her genes (…) but on the other hand, we are still facing uncertainties. What is the cause? Did something happen during pregnancy, or is it something genetic? (no diagnosis, interview 10) “I hope that technology will improve in such a way, that 1 day, we will receive a definitive diagnosis. But on the other hand, if she [the child] continues to develop the way she does now, then I think we do not need to know the cause because she is doing fine right now. And therefore, so do we. But when her situation worsens, and that might happen you know, then you do want to have a diagnosis in order to prepare yourself for what might happen.” (possible diagnosis, interview 19)
Loss of hope	“On the one hand you are relieved or even happy (…) because you know the cause, but on the other hand you become aware of all the things that are not possible anymore. It is like an emotional rollercoaster (…) you realize the situation [of the child] will not change. With that muscle disease we thought ‘Well, let us give him some medication and he will improve, and something like that will not happen now’.” (conclusive diagnosis, interview 9).
Disappointment about the lack of information	“When we heard it is not mitochondrial disease, you think ‘Yay, champagne!’ (…) but this new diagnosis is too vague right now. Not much is known yet and the information we did receive is very broad and general, like ‘It might be that…or it is possible that…’ (…) there is no written information on this syndrome (…) but I want to have proof. I want to show other healthcare specialists, ‘Look here, this is what is wrong with my child’ (…) you know, I want to move forward and arrange the care my child needs.” (conclusive diagnosis, interview 2) “There is a specialist on this disease, but he lives in the United States. That is a pity (…) I think scientists are not interested in this disease because it is so rare. But I hope that 1 day, more information will become available.” (conclusive diagnosis, interview 1) “I visited our family doctor and asked: ‘Do you know what is wrong with our child?’ ‘No’ was his answer. I did not receive any information about the diagnosis. And that annoys me you know, something is found, and he knows nothing about it.” (conclusive diagnosis, interview 2)
Parents dealing with new information	“I find it hard to deal with the results. I mean they told us that [next to mutation in DNA of child] also something is found, in my [the mother] X-chromosome, but we do not know for sure whether this mutation is the cause. Nobody in our family has this disease (...) so is it just accidental? We just do not know, and that is difficult to deal with. For now, they [the clinicians] cannot give us any further explanation.” (possible diagnosis, interview 20) “One of the parents we met at the conference developed a leaflet to raise awareness about this new syndrome. I ordered some copies and distributed that within our local hospital and day-care.” (conclusive diagnosis, interview 1, after 6 months)

### Caring Activities; Management of Care

Parents with a diseased child are perpetually confronted with a burden of care. Their daily care duties cover a wide range of activities, including nursing a disabled child, obtaining the necessary resources and equipment (e.g., a wheelchair), seeking specialized professional help to alleviate complaints, and administrative tasks such as keeping up to date with insurance regulations. These caregiving duties are demanding, and some parents described their engagement in them as a continuous “battle.” Because of the nature of this consuming work, some parents—mostly mothers—resigned from their jobs and others decided to work fewer hours. In some cases, having a diseased child also led to financial struggles as not all care-associated costs are reimbursed by either the healthcare system and/or government (e.g., adapting the house to their child’s wheelchair needs).

Parents in this study expressed hope that a diagnosis will improve care. In a few cases, the diagnosis indeed led to improvements such as referral to a different health professional, or support to arrange the necessary equipment.

One parent couple receiving a conclusive diagnosis initially felt empty-handed because of the lack of precise information on the prognosis and daily management; however, after 6 months they indicated the diagnosis and the neurologist’s referral to a rehabilitation physician enabled them to attune care facilities more closely to the needs of their child. Parents receiving no diagnosis also reported there were no changes in the daily management of their child’s care.

In cases where a definitive diagnosis did not lead to (major) changes or improvements in daily care, it did initiate shifts in the parents’ consideration of themselves as “good, responsible parents.” For example, one parent couple mentioned feeling insecure about their decisions regarding therapies for their child, because they felt judged by members of their social circle; to them the diagnosis was a confirmation that they were acting responsibly. After 6 months, two parent couples who had received conclusive diagnoses also mentioned having become more accepting towards the situation as it is. In contrast, two parent couples, respectively receiving a possible and no diagnosis, reported having to juggle between feelings of accepting and enjoying their child and still longing for a (conclusive genetic) diagnosis in the future.

Text box 2 Quotations that illustrate managing burden of care

**Table:** 

Changes in daily care management of the child	“The diagnosis did not really change things, but we have made an appointment with the ophthalmologist as the gene in which the mutation is found is linked to eye disease.” (Possible diagnosis, interview 18) “Based upon the WES test results, our rehabilitation physician initiated more therapy and prescribed leg splints for the night.” (possible diagnosis, interview 17) “Our neurologist referred us to a rehabilitation physician. That opened doors. We now have a wheelchair and a special needs bike for our child.” (conclusive diagnosis, interview 2, after 6 months)
Enhancing coping process	“We accepted the fact that not much is known about this syndrome. It is our task to make the best of this situation.” (conclusive diagnosis, interview 1) “I hope science will deliver answers, but in the meantime, we have to enjoy our child as much as possible.” (possible diagnosis, interview 19) “You know, a diagnosis does mean something (...) it does not provide a solution to our problems, our child will not get better. But for the coping process (...) you can come to terms with the situation (…) you see the difference (…) S. does actually have a [clinical] diagnosis, and yes, the fact that she has to use the wheelchair once in a while is more easy to accept for us, because indeed, long distances are too tiring for her (…) you accept that, why? Because it is allowed. Because S. has a [clinical] disorder [for which the cause has not been identified yet], and she really needs the wheelchair. But actually, N. [an affected sister of the diseased child who, at the time of the interview, had neither a clinical nor genetic diagnosis^a^] has the same problems [in mother’s opinion], but then you start thinking: ‘Why do you [N.] want to be in a wheelchair? Is it not just in your head?’ While really, she [N.] is so tired.” (no diagnosis, interview 8)
Confirmation of being a responsible parent	“And you feel uncomfortable… or you will be more harsh towards your child. Not because you want to, but to prove to others that you are also critical, and you know, you think about all these things. And now, when there is a diagnosis, it does, it gives you a feeling of “I told you so, we were right.” (conclusive diagnosis, interview 6) “That a gene mutation is found is a confirmation that it is not our fault. You know, we struggled with the idea that maybe we did not challenge him enough when he was a baby. And moreover, he fell off the changing unit once, and I always wondered whether this fall had anything to do with his disease.” (conclusive diagnosis, interview 9)

^a^N., the sister of the patient included in our study has meanwhile been seen by a clinical geneticist and has been diagnosed with a different disorder than her sister, for which the genetic cause has been identified

### Finding a Supportive Environment

Having a diseased child also influences the social life of parents. The extensive caring activities make it more difficult for parents to join in social activities outdoors, which sometimes leads to diminished friendships and other social contacts. Some parents established new social contacts, for example, through a patient organization, by meeting other parents at specialized care facilities, or through social media. Other parents, by contrast, mentioned not wanting to be part of a peer support community because they anticipated that only sorrows about the diseased child would be shared, while they wanted to highlight positive aspects.

Our interview data suggest that WES-results led to renewed feelings of isolation for some parents receiving conclusive and possible diagnoses. As WES produced a “new label” for the child’s disorder, some parents no longer felt at home in their patient organizations. Parents experienced this as a loss as they valued the exchange with—and support from—other parents within a patient organization.

Despite initial feelings of isolation, a conclusive diagnosis also enabled parents to establish new relationships with peers. After receiving the diagnosis, parents searched the internet for more information on the diagnosis and came across blogs, Twitter messages, and Facebook pages of other parents. Some of the parents contacted parents on the other side of the world via internet, whereas others reported that language was a hurdle for sharing their experiences. Moreover, some parents expressed not feeling an urge to establish contacts because symptoms may differ so much, even though children may have the same syndrome.

Text box 3: Quotations that illustrate experiences concerning peer relations

**Table:** 

Loss of peer support networks	“I was really active for [patient organization]. Yeah really focused. But now this moves to the background a bit. I think that is a pity. With regard to our new diagnosis, not many people have this disease. The neurologist told me that there are only two other patients with the same diagnosis here in the Netherlands.” (conclusive diagnosis, interview 1) I am no longer active on that forum, but I still read the stories of other parents once in a while. And it is still informative as we share the same concerns and struggles (…) but I feel, or I doubt, whether the other parents still want me there (…) I try not to respond as much as I did before because our child has a less severe prognosis now. And maybe the other parents do not like that.” (conclusive diagnosis, interview 5) “I could of course ask whether there are more children within our patient organization who have a mutation on the [name of gene], but there is no point in doing that. I already know that nobody knows this gene. That is the situation we have to deal with.” (possible diagnosis, interview 19) “We do not have a name of a syndrome for which we can fight, by raising funding for example. Other parents do. And then, yeah, then you have your back against the wall.” (possible diagnosis, interview 19)
Establishing relations with new peers	“I googled and there is indeed a forum, of course, very very small as it concerns a new syndrome, but I do not feel prompted to respond or share my story. I want to highlight the positive and the forum focuses on the negative aspects. (conclusive diagnosis, interview 2) “For this syndrome we found a forum hosted by people from the United States. I am not active [on the forum] as I find the English language difficult (.) The forum is useful to receive suggestions (...) and we see physical similarities between our child and theirs. Although seeing these similarities is not exactly a nice feeling, but that’s what it is.” (conclusive diagnosis, interview 5)

## Discussion

### Practice Implications

Parents’ evaluations of a genetic diagnosis, as found in this study, can provide insight into psychosocial aspects that should be taken into account in pre-test as well as post-test counselling. Though our study was carried out in a pediatric neurology setting, the findings offer some insight for other medical specialists who deal with WES test results and genetic counselling.

In agreement with previous research, our results show that genetic test results are not only a biomedical description to parents, explaining the genetic cause for malfunction or disease (De Ligt et al. 2012; Vissers et al. 2010). Parents also use genetic test results to deal with the social dimension of having a diseased child. In some cases, WES results offer, for example, a label for the condition of their child which allows parents to explain the child’s behaviour to the outside world, organize adequate care, and identify with a supportive group of peers (Eisenberg 1977; Hofmann 2002; Whitemarsh et al. 2007). We found, however, that the WES diagnosis did not always match the identity of the patient organizations the parents had joined in the past, and parents often no longer felt at home in their peer support groups. Consequently some parents mourned the loss of the support and friendships they had enjoyed there.

Parents receiving a possible diagnosis in particular, experienced a loss of peers as their “peers” still remain to be identified. Moreover, new uncertainties were evoked in cases where a presumable maternally inherited X-linked recessive mutation was identified. For those parents who received a conclusive diagnosis, one would expect that if WES is used as first-tier diagnostic test in routine clinical practice, it could enable parents to find adequate peer support more quickly and easily. Our results suggest, however, that a conclusive diagnosis will sometimes create difficulties. When WES identifies the cause of a rare disease or syndrome, peer support groups are often (still) small and international. The international aspect requires parents to express themselves in a foreign language, and some parents cannot exchange their experiences with peers or feel hesitant to do so because of the language barrier. Other parents with more proficiency in English and who received a conclusive diagnosis were enabled to connect with peers world-wide and share experiences via social media (see also Fanos 2012; Reiff et al. 2012).

Genetic counsellors should prepare parents for these difficulties before the test and continue to support them after test results have been obtained. What is important to keep in mind is that even when WES provides a diagnosis and ends the diagnostic odyssey, it may at the same time open up a new “odyssey.” Specifically, it may engage parents who received a conclusive diagnosis in a search for information about their child’s (very rare) disease and the establishment of new care arrangements, as well as a search for new peer contacts, which may be accompanied by (temporarily) mourning the loss of previous contacts. Furthermore, for parents who receive a possible diagnosis, or no diagnosis at all, there are little changes: they will continue to search for a diagnosis and will continue to be uncertain about the cause of their child’s disease until a confirmed diagnosis can be provided. Parents who receive a possible diagnosis, however, may in addition lose peer-contacts if the received lead for a diagnosis does not coincide with the identity of the patient organization they joined previously.

It is important that counsellors are aware of these uncertainties and losses that parents may suffer in their private lives, even if they receive the diagnosis for which they longed. While single gene testing also raises hopes as well as uncertainties, testing with WES will increasingly confront parents with diagnoses of very rare diseases about which little knowledge may be available. For counsellors this means they will have to inform and support parents, even though they may themselves have little to no previous experience with a disease (cf. Navon 2012). This may complicate the counselling process. Our research suggests that it may be advisable to prepare parents in advance for this continued uncertainty after obtaining the test results, and to provide them with follow-up care in order to deal with the concerns and questions raised by the results (Krabbenborg et al. 2015). Furthermore, counsellors can re-contact parents and inform them when new literature about their child’s disease becomes available in order to diminish their uncertainty (see also Reiff et al. 2012). Counsellors can also help parents connect with new peers around the globe. Different from targeted genetic testing, WES has the intrinsic risk of the identification of incidental or secondary findings. Our study has shown that the identification of potential diagnostically relevant sequence variants in the parents (e.g., X-linked recessive mutations in mothers) leads to new questions and uncertainties. Yet, we have previously shown that the desire of parents to identify the cause of their child’s disease nullifies any pre-test distress on the potential risk of identifying any medically relevant genetic variants in their own DNA (Krabbenborg et al. 2015). This combination of factors indicates that sufficient amounts of time should spent in pre- and post-test counselling to ensure parents understand the potential risks of the test and how such potential risks may affect their lives.

### Study Limitations

Several factors may have influenced our results. Firstly, we recruited respondents from one (tertiary) hospital, in one country (and thus one type of healthcare system). In addition, the vast majority of all interviewees (95 %) were of Caucasian origin. Therefore, our results may not be representative for families worldwide using WES as a diagnostic test. Second, while it is a strength of our study that we included all three types of test results that can be obtained using WES (i.e., a conclusive, possible, or no genetic diagnosis), enabling us to make a comprehensive comparison of the experiences obtained in these groups, we did not initiate follow-up interviews with parents receiving no diagnosis. This creates limitations in comparing longer-term experiences. Third, the small samples for each groups make it difficult to draw conclusions about thematic differences due to type of result received. Fourth, there was variability in time regarding carrying out the interviews. This might have influenced the results; for instance, parents from group 2 (possible diagnosis) were interviewed 2 to 6 months after they received the test results and thus had more time to experience how the WES test results influenced their daily life than parents from group 3 (no diagnosis) who were interviewed within 1 to 2 months after they received the test results. Finally, as some interviews were conducted by telephone, this may have influenced the results through the absence of non-verbal cues; additional probing questions aimed at acquiring more insight, in response to certain types of non-verbal behaviors, are not possible on the telephone.

### Research Suggestions

A study with more respondents across hospitals and nations would be worthwhile as it would allow for deeper insight into, for instance, whether and how culture, class and different healthcare systems influence parents’ experience WES test results in daily life. Secondly, longitudinal research into all three types of WES test results (conclusive, possible, no genetic diagnosis) would be useful to monitor how parents cope with WES test results over a longer period. It may, for instance, be expected that patients receiving a possible genetic diagnosis will in the future receive a definitive one when additional patients are identified. Moreover, the increased use of WES may allow for identifying more children with the same rare diseases and syndromes in the near future, which is likely to alter the feelings of mourning and isolation that were expressed by some parents who lost their peer group.

## Conclusions

Whereas WES has rapidly founds its way into daily genetic diagnostic care, empirical data on the experiences of patients and/or parents on how to deal with the WES results in their daily life are still lagging behind. Our exploratory study aimed to provide some of this information. Our main conclusion is that WES test results evoked ambivalent responses, irrespective of the type of test result they obtained. Specifically, a conclusive diagnosis enabled parents to 1) become more accepting towards the situation; 2) cope with feelings of guilt; 3) deal with the outside world; and 4) attune caregiving activities to the child’s needs. In addition, a conclusive diagnosis confirmed for parents that they were on the right track with regard to arranging therapies for the child. Parents receiving a possible diagnosis also used the diagnosis to adjust caring activities more to the needs of the child. However, a diagnosis could also elicit new worries, such as in the case of the potential X-linked mutations which were identified in the mother. Parents receiving no diagnosis vacillated between accepting that they have to continue living with uncertainty and longing for a continuation of research in order to receive a diagnosis at a later stage. Whereas most of these experiences cohere with previous studies in the context of rare genetic syndromes (Bosma et al. 2015; Brooks-Howell 2006; Graungaard and Skov 2006; Hallberg et al. 2010; Reiff et al. 2012; Skirton 2001; Webb 2005), they have not been studied in a largely unselected patient cohort with a presumed genetic disease. For example, the likelihood of these patients receiving a genetic diagnosis using WES was much greater than when testing for genetic syndromes by gene-specific Sanger sequencing tests. In addition, the potential for WES to result in “possible” diagnoses, where further research is needed for confirmation, may pose new uncertainties, and our study yielded some initial insights into how parents deal with them.

## Footnotes

1 One interview was carried out by author SvdB
